# CNN‐Based Neurodegenerative Disease Classification Using QR‐Represented Gait Data

**DOI:** 10.1002/brb3.70100

**Published:** 2024-10-28

**Authors:** Çağatay Berke Erdaş, Emre Sümer

**Affiliations:** ^1^ Department of Computer Engineering, Faculty of Engineering Başkent University Ankara Turkey

## Abstract

**Purpose:**

The primary aim of this study is to develop an effective and reliable diagnostic system for neurodegenerative diseases by utilizing gait data transformed into QR codes and classified using convolutional neural networks (CNNs). The objective of this method is to enhance the precision of diagnosing neurodegenerative diseases, including amyotrophic lateral sclerosis (ALS), Parkinson's disease (PD), and Huntington's disease (HD), through the introduction of a novel approach to analyze gait patterns.

**Methods:**

The research evaluates the CNN‐based classification approach using QR‐represented gait data to address the diagnostic challenges associated with neurodegenerative diseases. The gait data of subjects were converted into QR codes, which were then classified using a CNN deep learning model. The dataset includes recordings from patients with Parkinson's disease (*n* = 15), Huntington's disease (*n* = 20), and amyotrophic lateral sclerosis (*n* = 13), and from 16 healthy controls.

**Results:**

The accuracy rates obtained through 10‐fold cross‐validation were as follows: 94.86% for NDD versus control, 95.81% for PD versus control, 93.56% for HD versus control, 97.65% for ALS versus control, and 84.65% for PD versus HD versus ALS versus control. These results demonstrate the potential of the proposed system in distinguishing between different neurodegenerative diseases and control groups.

**Conclusion:**

The results indicate that the designed system may serve as a complementary tool for the diagnosis of neurodegenerative diseases, particularly in individuals who already present with varying degrees of motor impairment. Further validation and research are needed to establish its wider applicability.

## Graphical abstract

The study proposes a CNN‐based method using QR‐represented gait data for neurodegenerative disease classification. The approach effectively distinguishes between different gait patterns, highlighting the potential of QR encoding in enhancing CNN classification performance for medical diagnostics.

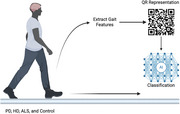

## 1 Introduction

Throughout their lives, people seek the advice of medical professionals and employ a range of medical techniques to maintain physical and mental well‐being and to address the ailments they experience. Neurology is a medical specialty concerned with the anatomical structure, functions, and abnormalities of the nervous system. It encompasses a wide range of diseases from neurodegenerative disorders to stroke (Erdaş, Sümer, and Kibaroğlu 2021).

Movement disorders are among the most prevalent neurological disorders (Huang et al. 2023). While movement disorders manifest as hypokinetic or hyperkinetic disorders, some subtypes are specific to the disorder experienced by the patient in both areas. Parkinson's disease (PD) is a neurodegenerative condition that develops when brain cells are lost prematurely, and it is one of the most illustrative examples of this phenomenon. While there is currently no treatment that can prevent or halt the pathophysiological processes associated with the disease, there are several symptomatic treatments that can be employed. In PD, it is of the utmost importance to maintain continuous monitoring of the symptoms of the disease, including trembling (tremor), slow movement (bradykinesia), involuntary muscle contractions (dyskinesia), and gait disturbances. Based on these observations, it is crucial to adjust the dosage of medication accordingly. Therefore, the quality of life of the patient is enhanced. Parkinson's disease affects approximately 1% of the global population and progresses with the worsening of the symptoms mentioned above as the patient ages (Albanese et al. 2013; Kaleağası & Dogu 2015).

Huntington's disease is a fatal autosomal dominant disorder typically seen at the beginning of adult life, manifesting in its progressive course as a mixture of cognitive, behavioral, and motor symptoms. The disease manifests between the age of 35–50 years, and the life span after the first diagnosis is 15–20 years (Bates et al. 2015). The beginning of Huntington's disease is defined as the onset of motor symptoms, and often the first signs leading to patients seeking medical treatment are clumsiness, tremors, loss of balance, or concussion. The most important representative movement abnormality and typically the earliest symptom; the persistent movement of writhing and jerking, also known as chorea or choreoathetosis. The most prominently affected parts are the limbs and the trunk but may include the respiratory and muscular systems (Winder et al. 2015).

The neurons in the brain and spinal cord are impacted by the progressive neurodegenerative disease known as amyotrophic lateral sclerosis (ALS) (Wu and Sin 2010). From the brain to the spinal cord, motor neurons commute to the muscles of the body. Gradual ALS nerve cell deterioration could lead to the patient's death. Once nerve cells die, the brain's capacity to activate and regulate muscular activity is destroyed. ALS is a gradual disease that principally impacts nerve cells in the indirectly associated muscle movements, as well as the spinal cord, brain stem, and cerebral cortex (Sengar, Dutta, and Travieso 2017). When these muscle movements are gradually impacted, people may lose their ability to eat, speak, breathe, and move. ALS is a disease that usually occurs between the age of 40 and 70 years (Tomik & Guiloff). The disease may progress to a size where symptoms worsen with a period. There is reportedly no cure for ALS, and no successful therapy is available to slow or reverse the disease's progression. The early signs of ALS usually manifest as fatigue or rigidity in the muscles. All muscles are slowly impaired, and individuals lose their energy, and their ability to eat, speak, breathe, and move. Symptoms may be so infrequent and low in severity at the onset of ALS that they may be neglected, but these symptoms may gradually progress to a more pronounced weakness or atrophy that may cause a doctor to suspect ALS (Pham 2017). The first sign of ALS may appear in the hand or arm, while another is when the legs are affected. People complain of being clumsy in their daily handicrafts, of being awkward while walking or running, and of stumbling more often (Bakker et al. 2020).

This study hypothesizes that gait data when transformed into QR codes and classified using convolutional neural networks (CNNs) can effectively discriminate between neurodegenerative diseases such as PD, Huntington's disease (HD), and ALS compared to a control group. The approach assumes that encoding gait data as QR codes provides a two‐dimensional (2D) representation that can enhance CNN's ability to recognize and classify disease‐specific gait patterns. It is expected that this novel method will provide accurate classification results, thus providing a complementary tool for the diagnosis and differentiation of neurodegenerative diseases based on gait analysis.

## 2 Related Works

Manap, Tahir, and Yassin (2011) analyzed the parameters that can be used to identify the unusual movement patterns in Parkinson's disease. Three types of parameters of gait, fundamental, kinematic, and kinetic, have been assessed. The initial results of the analysis showed that Parkinson's patients had lower average speed, gait acceleration, and stride length than the control group and that PD patients had a higher average wait time. On the other hand, in the context of kinematic parameters, mean values of general joint hip angle, knee, and ankle were found to be lower in Parkinson's patients than in the normal population. In addition, it was observed that all average values of the main reaction force parameters were higher in the control group, where the growth rate was the most effective determining factor for the kinetic parameter. Statistical analysis was performed to evaluate important characteristics that could be used as an identity between Parkinson's patients and normal subjects. The use of artificial intelligence for the classification problem was carried out using vertical properties of ground reaction force, walking speed, and stride length. Lee and Lim (2012) studied the classification of PD by using data on gait. They extracted wavelet attributes to classify patients from the gait signals they gathered from ground reaction force (GRF) sensors. They proposed an artificial neural network structure with weighted fuzzy membership functions (NEWFM) to adhere individuals with PD and control group. Daliri (2013) aimed to use the features obtained from GRF signals by short‐time Fourier transform to detect PD. A chi‐square distance‐based DVM kernel was chosen for the classifier. Ertuğrul et al. (2016) calculated local binary patterns (LBP) from the walk signal for the representation of the feature. They generated statistical features using LBP histograms. They created the final set of attributes using a feature selection method that is correlation based. The most successful classification performance was obtained with the multi‐layer perceptron classifier. A two‐level classifier was developed by Zeng et al. (2016) to distinguish PD from the control group using gait data. From walking signals to use in classification, they extracted four attributes. These attributes involve the variations between the output of certain sensors located below both feet. They classified the feature vectors using an artificial neural network that uses a radial basis function kernel. Açıcı et al. (2017) suggested a random forest‐based method for Parkinson's disease identification. To feed the random forest classifier, they extracted frequency‐based and time‐based attributes from the walking signals. They stated that it is possible to achieve higher precision compared to previous methods in the literature. Aşuroğlu et al. (2018) developed a model of locally weighted random forest regression (LWLRF), tracking the motor symptoms of PD. They stated that the local weighting scheme they suggested helped counteract the effects of variability in gait patterns.

Hausdorff et al. researched the time‐series data about the step interval of walking in people with Huntington's disease. The degree of correlation between a step interval and the preceding and following intervals was measured temporally. Their results indicate that shifts in phase intervals were more spontaneous in Huntington's disease patients than in the control group. Bellotti et al. (2004)studied in Huntington's disease using EEG patterns. An artificial neural network fed by the alpha rhythm of brain waves was used. Mannini et al. (2016) proposed a general probabilistic modeling approach to classify different pathological gaits in their study. The proposed technique used gait data recorded from two populations of patients diagnosed with neurodegenerative diseases, Huntington's patients and patients with stroke, and inertial measurement units placed at the waist. Features were obtained via hidden Markov models (HMMs) and besides that, the features extracted in the time and frequency domain were classified into a support vector machines classifier (SVM). As a result, the classification success was measured as 90.5%. Huang et al. (2019) aimed to develop a machine‐learning technique to predict disease by gait information from Huntington patients. The relevant approach includes extraction of features that are relatively less used in the literature, feature selection, parameter adjustment, and cross‐validation steps. Bennasar et al. (2016) proposed an innovative method to differentiate accelerometer data from control and HD individuals. For this, the proposed technique used signal processing during preprocessing, feature selection methods to determine parsing features, and finally various classification techniques. From the data obtained from three different accelerometer sensors, 90 time‐space features were extracted, thus it was planned to capture the disease‐specific pattern.

Hausdorff et al. (1997) in patients with ALS, the extent of stride fluctuations and gait rhythm is taken into account relative to the control group. In addition, in people with PD and HD, these criteria have also been studied. It was observed that, relative to healthy subjects, ALS patients had restricted gait movement. Overall, in all three classes of neurological disorder, stepwise variability and synchronicity increased relative to stable healthy people. Wu and Sin (2010) aimed to develop a method of analytical study to identify ALS patients and control group gait results. Walking probability density functions have been attempted to be estimated in their strategy. With this approach, ALS patients and the control group were able to identify phase patterns with an 82.8 percent success rate. Bilgin and Akın (2018) focused on detecting ALS patients in their study. For this, they extracted the features by using the discrete wavelet transform applied to the walk. They fed various machine learning algorithms with the extracted features. Alaskar and Hussain (2018) utilized gait data to detect and classify ALS disease with its attributes and various classifiers, such as the linear and quadratic discriminant classifiers. They discussed whether they could distinguish between cases of sclerosis disease.

To improve the accuracy, Yang et al. (2009) examined three distinct feature selection techniques: signal‐to‐noise ratio (SNR), maximum signal‐to‐noise ratio (MSNR), and principal component analysis (PCA). Baratin et al. (2015) found it useful in classifying control and patients with neurodegenerative diseases using wavelet transform to generate differential properties for coherence and empirical examination of entropy. Gupta et al. (2019) studied autocorrelation and cross‐correlation between walking data and time series, and a data‐based approach. In addition, they developed a decision tree–based classification application to differentiate neurodegenerative diseases from the control group.

Related works took advantage of classical machine learning and feature manipulation methods to solve the problem of classifying neurodegenerative diseases with the data obtained by sensors. More innovative solutions can be produced for the problem of classifying neurodegenerative diseases by using deep learning techniques such as the CNN. This is achieved through the comprehensive exploitation of the pattern recognition functionality intrintrinsic to CNN methodology. Futhermore, the data obtained via sensor deployment can be presented in two dimentions, facilitating enhanced comprehension of the underlying features.

In the present study, sensor‐based gait data collected from individuals with neurodegenerative disorders such as Parkinson's, Huntington's, ALS, and the control group were transformed into QR codes and classified by CNN deep learning method. The reproduction of gait data with QR codes and the usage of QR codes as an attribute in the classification problem with CNN is one of the novel aspects of this publication.

## 3 Materials and Methods

### 3.1 Dataset

**FIGURE 1:**
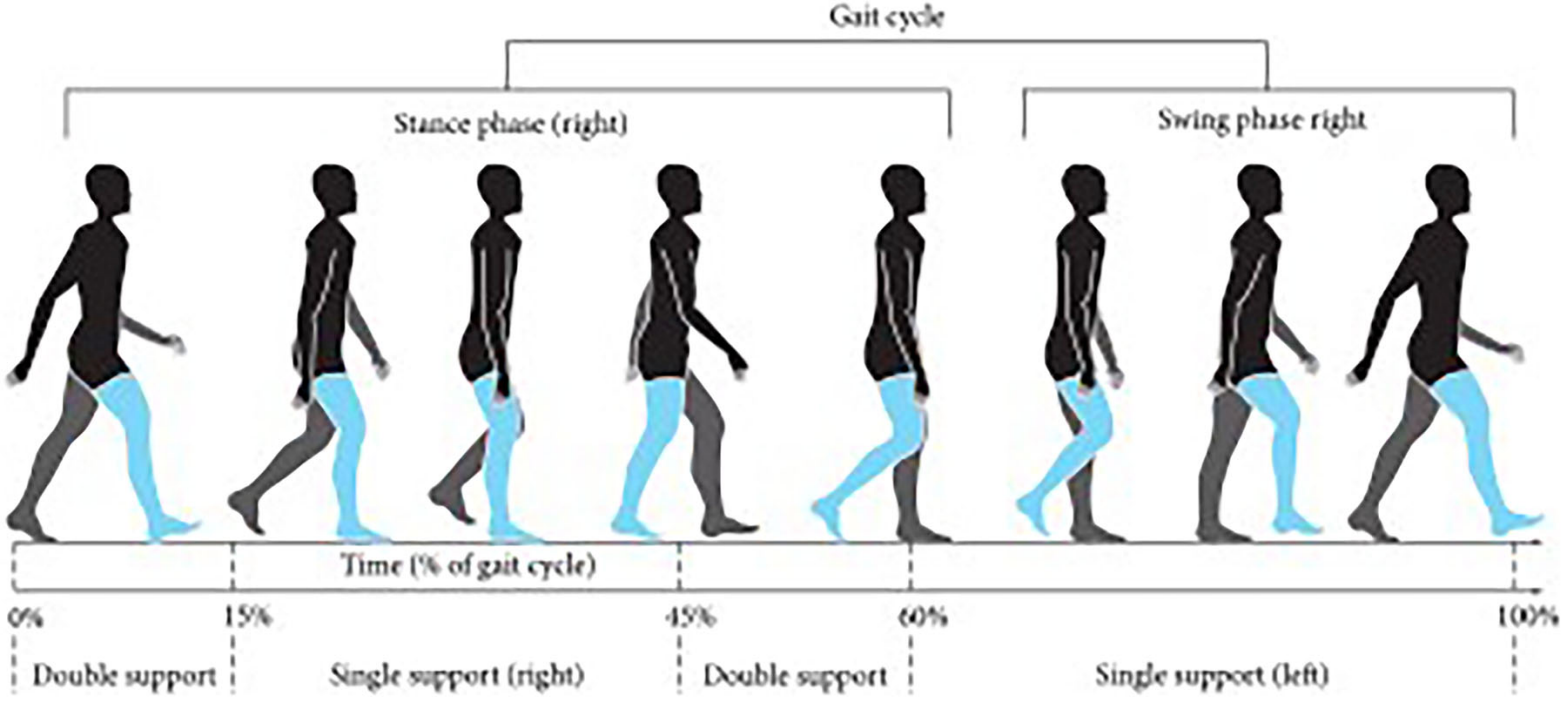
The illustration of gait phases.

The Physionet provided the dataset that was utilized, which includes gait signals of patients with neurodegenerative diseases (Hausdorff et al. 1997). The raw data were collected utilizing force‐sensitive resistors, which generated output that correlated with the force exerted on the foot. By analyzing these indicators, stride‐by‐stride measurements of the instances of footfall contact were determined. Ultimately, a total of 13 distinct features were collected from this process. The mentioned features are as follows: duration, left step timing, right step timing, left swing timing, right swing timing, ratio of left swing to left step, ratio of right swing to right step, left stance time, right stance time, left stance to left step, right kick to right ratio of step, duration of double leg support, and time of double leg support by step. In this study, only 12 features were considered and the duration attribute, which provides information about time steps, was excluded from the analysis. The 12 features chosen for this study are highlighted in Figure 1 and relate specifically to the right leg. The same process and criteria used to identify and sum these features were also applied to the data collected from the left leg.

### 3.2 General Framework

**FIGURE 2:**
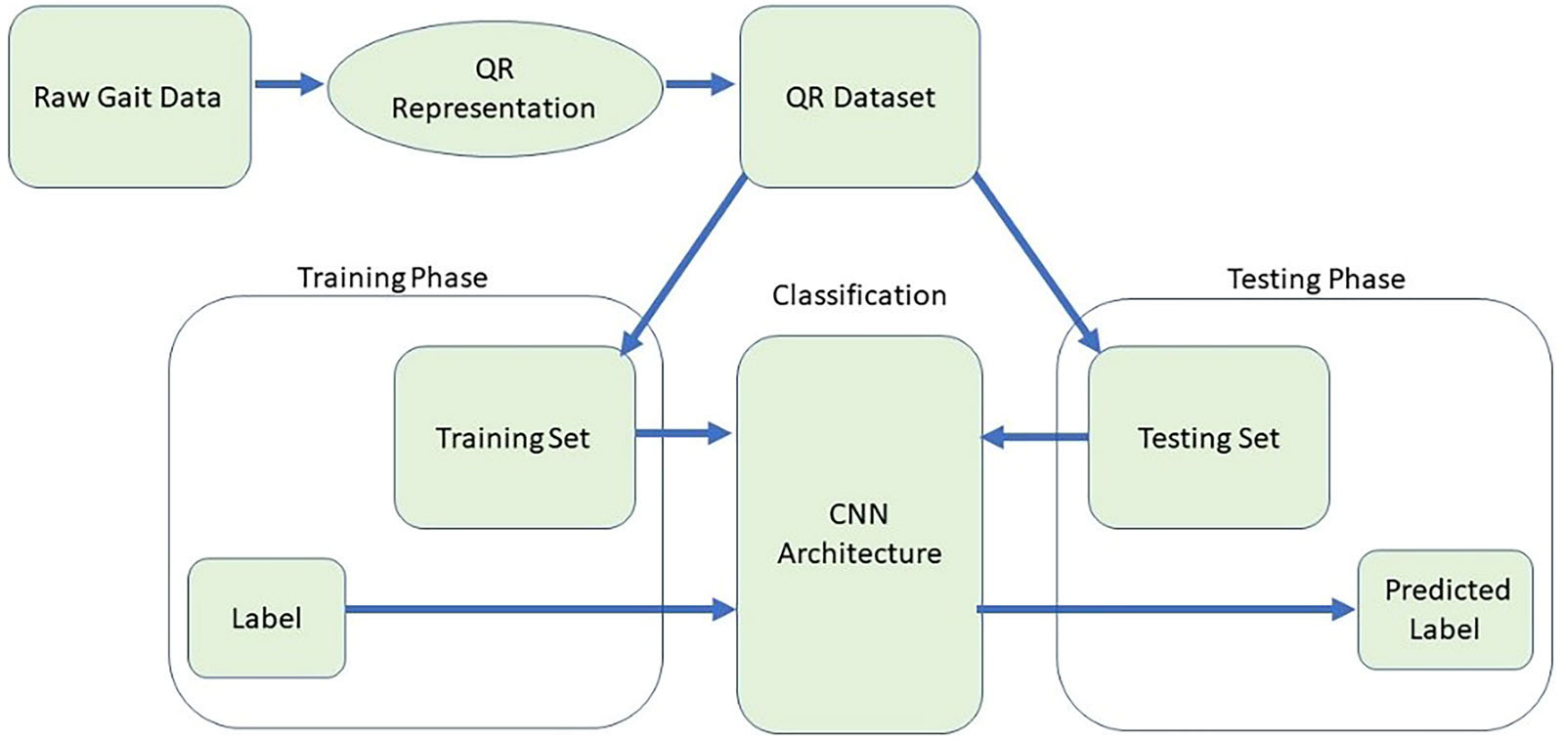
The proposed method's general framework.

The outline of the proposed method is shown in Figure 2. The samples in the new QR code dataset fed into the proposed CNN architecture sought solutions to these neurodegenerative disease classification problems. The problem of classifying the relevant numerical data has been turned into a problem of classifying QR patterns.

**FIGURE 3:**
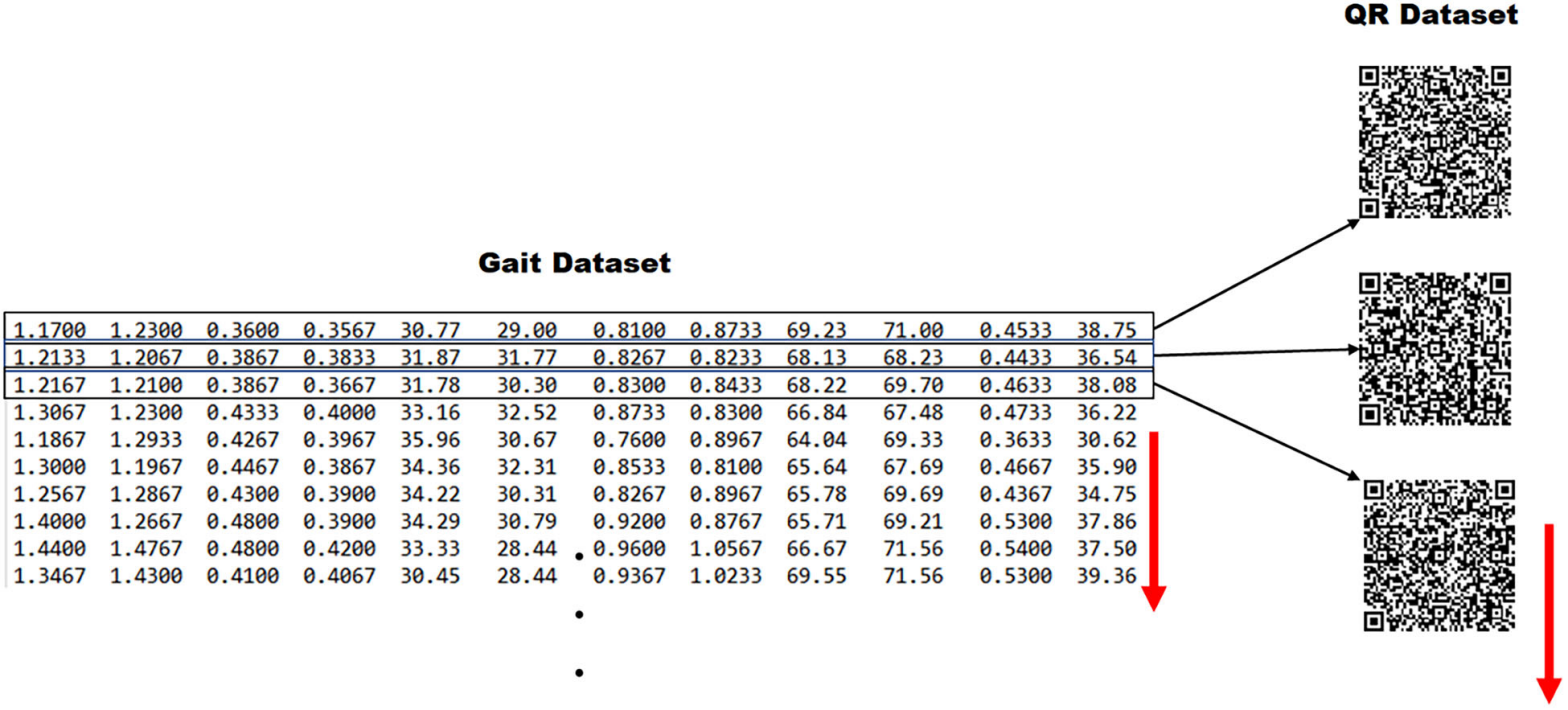
QR dataset creation.

The dataset, which consisted of 15,092 samples (2550 from ALS patients, 4846 from HD patients, 3620 from PD patients, and 4076 from the control group), was transformed into QR codes of 100 × 100 pixels using the version 10 standard. This resulted in the creation of a new QR code dataset. To ensure accuracy, the error correction parameter, denoted as “*m*”, was also specified when using the version 10 standard. The process of creating the QR code dataset is depicted in Figure 3.

### 3.3 Convolutional Neural Network

CNNs are a popular form of deep learning algorithm that is used to learn from images, videos, text, or audio for classification tasks. These networks are particularly useful for finding and identifying patterns that often occur in images (Sainath et al. 2019).

**FIGURE 4:**
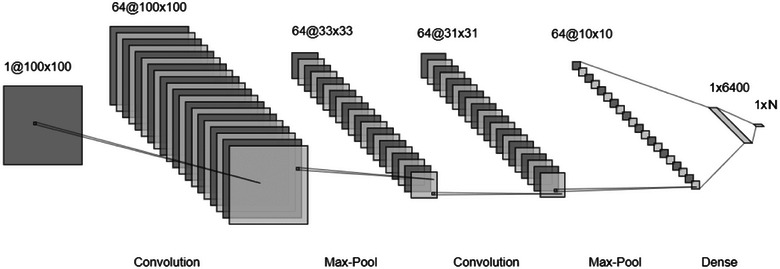
The CNN architecture used for this study.

The architecture of the CNN used in this study is illustrated in Figure 4. The process involved transforming each 100 × 100 single‐channel QR code in the QR dataset into 64 feature maps of 10 × 10 using the layout depicted in Figure 4, followed by classification with a dense layer at the end. To elaborate, the architecture begins with a convolutional layer, which was used to generate 64 feature maps without altering the original input dimensions. These feature maps, which had a size of 64 × 100 × 100, were then reduced to 64 × 33 × 33 by using a max pooling layer. This feature map of 64 × 33 × 33 dimensions was further reduced to 64 × 31 × 31 and 64 × 10 × 10 with subsequent convolutional and max‐pooling layers, respectively. The architecture also includes two different dense layers with 6400 units and *N* units, where the value of *N* changes depending on how many categories there are in the problem, and this can be two or four.

## 4 Results

### 4.1 Performance Evaluation

The experiments utilized a ten‐fold cross‐validation method in which the dataset was divided into 10 equal portions, each one containing a balanced number of samples from every category. In this method, one portion was utilized in testing while the others were utilized in training, and this procedure was repeated until each portion was utilized in testing. The system's ultimate performance was reported as the average of the classification performances across all portions (Han et al. 2020). Additionally, metrics such as accuracy, precision, sensitivity, and F1 score were used to evaluate the classification performance of the approaches.

The accuracy of a model is a measure of its ability to correctly classify examples in a classification problem. It is calculated as the proportion of correctly classified examples to the total number of examples, as shown in Equation (1) (Ölçer and Temizel 2021).

(1) $$\mathrm{Accuracy}=\left(\mathrm{TP}+\mathrm{TN}\right)/\left(\mathrm{TP}+\mathrm{FP}+\mathrm{TN}+\mathrm{FN}\right)$$

Precision refers to the ability of a classifier to avoid labeling negative samples as positive. Sensitivity, on the other hand, is a measure of a classifier's ability to correctly identify all positive samples. Equation (2) provides the formula for precision, and Equation (3) provides the formula for sensitivity (Erdaş et al. 2016).

(2) Precision=TP/(TP+FP)

(3) Sensitivity=TP/(TP+FN)

The F1 score combines the model's precision and sensitivity, and is described as the harmonic mean of model accuracy and sensitivity (Denck et al. 2019). F1 score's formula can be seen in Equation (4).

(4) $$F1\;\mathrm{score}=2\times \left(\mathrm{Precision}\times \mathrm{Sensitivity}\right)/\left(\mathrm{Precision}+\mathrm{Sensitivity}\right)$$

TP, TN, FP, and FN represent the number of cases correctly classified as positive, the number of cases correctly classified as negative, the number of cases identified as false positive, and the number of cases falsely identified as negative, respectively.

### 4.2 Empirical Results

Gait data are classified by the CNN deep learning algorithm after being represented by an image by converting it into QR codes. The classification success of the related method based on disease detection is shown in Table 1. Accordingly, the classification performance of four different groups/labels (ALS, Parkinson, Huntington, control) in the dataset of the proposed method was measured as 84.65% for accuracy metric, 90.56% and 80.14% for precision and sensitivity, and 83.98% for F1 score. All neurological diseases were collected under a single label and the classification performance of two separate groups as control and patient was measured as 94.86% for accuracy metric, 94.90% and 94.89% for precision and sensitivity, and 94.84% for F1 score. The classification performance of only ALS patients and the control group was 97.65% for the accuracy metric, 97.63% and 97.64% for the precision and sensitivity, and 97.62% for the F1 score. The classification performance of only Huntington patients and the control group was measured as 93.56% for accuracy metric, 93.58% and 93.55% for precision and sensitivity, and 93.56% for F1 score. The classification performance of only Parkinson patients and the control group was measured as 95.81% for accuracy metric, 96.04% and 96.38% for precision and sensitivity, and 96.04% for F1 score. Accordingly, in the experiments conducted with the proposed method, the most successful classification result was obtained in the ALS versus control sub‐problem with 97.65%. Furthermore, the classification results obtained for PD versus control, All NDD versus control, and HD versus control sub‐problems cannot be ignored either. The rate of accuracy obtained in four group sub‐problems in which all diseases and the control group are included as a separate class was measured as 84.65%. This result can be accepted as successful if it is taken into consideration that the diseases in different groups make the classification problem difficult. Also, the comparison of the related method with the studies performed on the same dataset in terms of the accuracy metric is shown in Table 2. None of the previous studies worked on four group sub‐problems.

**TABLE 1:** The classification performance of the proposed method.

	Accuracy (%)	Precision (%)	Sensitivity (%)	F1 Score (%)
4 Groups (ALS vs. HD vs. PD vs. control)	84.65	90.56	80.14	83.98
All NDD (ALS + HD + PD) vs. control	94.86	94.90	94.89	97.65
ALS vs. control	97.65	97.63	97.64	97.62
HD vs. control	93.56	93.58	93.55	96.04
PD vs. control	95.81	96.04	96.38	96.04

**TABLE 2:** A comparison of the proposed method to other researches.

Accuracy (%)	Zenget al. (2016)	Daliri (2013)	Yang et al. (2009)	Baratin et al. (2015)	Gupta et al. (2019)	This Study
4 Groups (ALS vs. HD vs. PD vs. control)	—	—	—	—	—	84.65
All NDD (ALS + HD + PD) vs. control	93.75	90.63	86.85	80.4	87.5	94.86
ALS vs. control	89.66	96.79	93.96	86.2	96.2	97.65
HD vs. control	83.33	90.28	84.17	86.1	88.5	93.56
PD vs. control	87.1	89.33	86.85	87.1	92.3	95.81

## 5 Discussion

Gait data from individuals with various neurodegenerative diseases, along with a control group, were transformed into QR codes for this study, an innovative representation approach. These QR codes were then analyzed using a CNN deep learning algorithm to assist in diagnosing neurodegenerative diseases. By converting the data into QR codes, a second dimension was added, making the data compatible with 2D CNN analysis.

The classification performances achieved in this study are noteworthy. Specifically, for the classification of four groups (ALS vs. HD vs. PD vs. control), the accuracy reached 84.65%. For other sub‐problems, including All NDD (ALS + HD + PD) versus control, ALS versus control, HD versus control, and PD versus control, the accuracies were 94.86%, 97.65%, 93.56%, and 95.81%, respectively.

The relatively lower classification performance for the four groups' sub‐problems, compared to other binary classification tasks, can be attributed to the complexity of distinguishing between multiple neurodegenerative diseases simultaneously. In contrast, binary classification sub‐problems benefit from a simpler evaluation based on the presence or absence of a disease. The lack of existing studies addressing the four groups' sub‐problems further supports the uniqueness and value of this study's approach.

Comparing this study with other research is challenging due to the absence of comparable solutions for the four groups' sub‐problems in the literature. This distinction highlights a significant contribution to this study. Additionally, for other sub‐problems, the proposed approach outperforms the best results reported in existing studies by margins of 0.86–3.51 percentage points. These findings suggest that the proposed method is not only generally successful but also holds promise for application to other datasets.

## 6 Conclusions

In this study, the gait data of patients with neurodegenerative diseases and the control group were transformed into QR codes, which is a novel representation, and classified with the CNN deep learning algorithm to diagnose the neurodegenerative diseases. The gait data used within the scope of this study were transformed into QR codes and represented by an image. In this manner, the raw gait data had been transformed into two dimensional representation. Gait data represented in two dimensions was sought to solve the problem of diagnosing the disease by feeding the CNN deep learning algorithm, which is a classifier that has promising results for 2D data. Moreover, such an approach has not yet been encountered in the literature.

Considering the results obtained for four groups (ALS vs. HD vs. PD vs. control), All NDD (ALS + HD + PD) versus control, ALS versus control, HD versus control, and PD versus control subproblems. The results achieved accuracy ranging from 93.56% to 97.65% for a single disease versus control group. The success of the classification achieved for the sub‐problem in the control group, in which all diseases were collected under a single label, was measured as 94.86%. The classification success for the sub‐problem with three different diseases and control groups is around 84.65%. In this context, with this study, a decision support system that helps physicians can be created, which (i) can predict whether the person has a neurodegenerative disease and (ii) if there is a disease, make a prediction about what this disease is.

Moreover, it is expected that the proposed QR representation method will apply to other research topics. Consequently, by transforming one‐dimensional data into two dimensions, the 2D CNN deep learning method can be employed, which offers advantages such as pattern capture and feature learning. Furthermore, as the QR transformation represents the data as a binary, this structure can be fed into a feature extractor comprising image metrics, which can then be utilized with machine learning algorithms for classification and regression. It would be beneficial for future studies to aim to expand the dataset to include data related to Alzheimer's disease. Such an expansion would facilitate a more comprehensive analysis and classification of major NDDs. It is anticipated that the inclusion of Alzheimer's disease data would enhance the robustness and generalizability of the model, thereby facilitating a deeper understanding of NDD classification. Also, future studies, it is intended to explore the classification of the datasets using other neural network‐based algorithms, such as generative adversarial networks.

## Author Contributions

**Çağatay Berke Erdaş**: conceptualization, investigation, funding acquisition, writing–original draft, visualization, validation, methodology, writing–review and editing. **Emre Sümer**: supervision.

### Peer Review

The peer review history for this article is available at https://publons.com/publon/10.1002/brb3.70100.

## Ethics Statement

As the dataset used in the study was created by other scientists and made available as an open source, ethical approval is the responsibility of those who contributed the dataset to the literature. Furthermore, their relevant studies have ethical approval by the Helsinki Declaration.

## Data Availability

The data that support the findings of this study are openly available in Gait in Neurodegenerative Disease Database (gaitndd/1.0.0) at https://physionet.org/content/gaitndd/1.0.0/.
